# 549. Monoclonal Antibody Therapy for COVID-19 Infection in Michigan: The Flint Experience

**DOI:** 10.1093/ofid/ofab466.748

**Published:** 2021-12-04

**Authors:** Mariam Younas, Danielle Osterholzer, Brandon R Flues, Carlos Rios-Bedoya, Philip McDonald, Ghassan Bachuwa, Michael W Tupper, Michael Jaggi

**Affiliations:** 1 Palmetto Health USC, Columbia, SC; 2 Hurley Medical Center, Flint, MI; 3 Hurley medical center, Flint, Michigan; 4 Hurley Medical Center / University of Michigan Medical School, Flint, Michigan

## Abstract

**Background:**

Bamlanivimab (BAM), a neutralizing IgG1 monoclonal antibody (mAb), received emergency use authorization (EUA) by the U.S. Food and Drug Administration (FDA) for treatment of mild to moderate COVID-19 infection in patients 12 years of age and older weighing at least 40 kg at high risk for progressive and severe disease on Nov 10, 2020. The purpose of this study is to describe our experience with this treatment modality.

**Methods:**

Hurley Medical Center (HMC), is a 443-bed inner city teaching hospital in Flint, MI. HMC administered its first BAM infusion on Nov 19, 2020. Through April 30, 2021, 407 patients with confirmed SARS-CoV-2 infection, received a mAb infusion. 62/407 patients received the combination mAb therapy of BAM + Etesevimab, as the EUA for BAM monotherapy was revoked on 04/16/21. We retrospectively collected basic demographic data and hospitalization to our facility within 14 days of receiving mAb therapy on these patients.

**Results:**

During the 5.5 month study period, patients receiving mAb therapy at HMC had a mean age of 56 years (yrs) (± standard deviation) (± 15.4) and a mean Body Mass Index (BMI) of 34 kg/m² (± 8.5) (Tables 1,2). African Americans (AA) comprised 48% (194/407) (Table 3) and females comprised 54% (220/407) of the cohort. 6% (25/407) of the patients required hospitalization within 14 days of mAb infusion, had a mean age of 58 yrs (± 17) (p-value 0.62) and a mean BMI of 32 kg/m² (± 9) (p-value 0.33). Females and AA comprised 56% (14/25) and 48% (12/25) of this subgroup respectively (p-value 1.0). No deaths were reported within 30 days of infusion in this cohort.

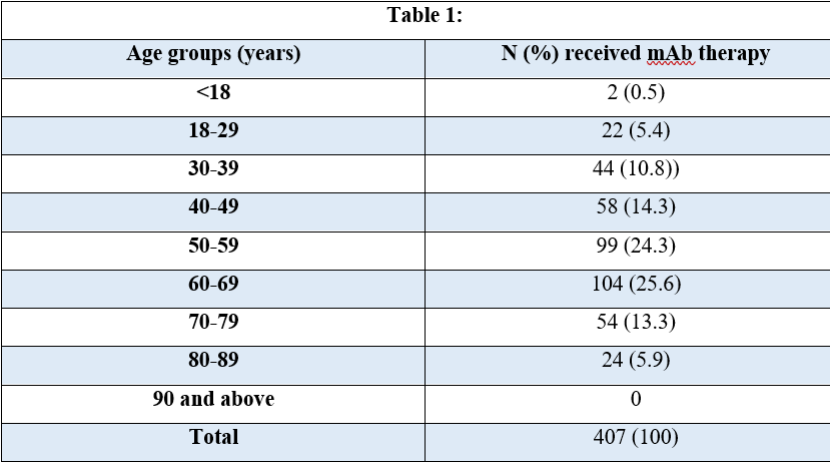

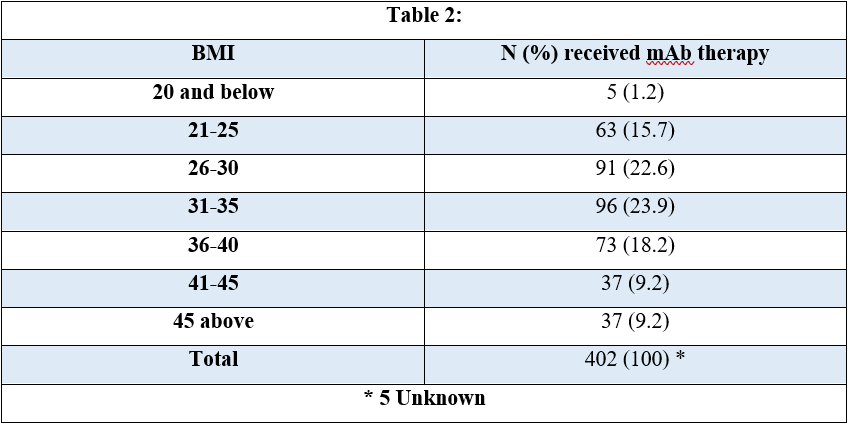

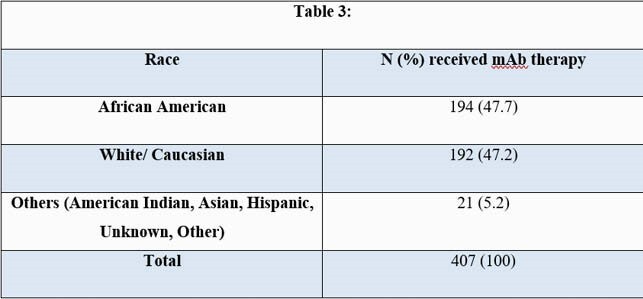

**Conclusion:**

Previously published reports cite a hospitalization rate in untreated high-risk COVID-19 infected patients of 9-15%. During the period of study, the county hospitalization rate and county mortality rate for all comers with COVID-19 was 6.6% and 2.7% respectively while our high risk cohort had a hospitalization rate of 6% and with no deaths reported. Our cohort had much lower rates of hospitalization and death than would be expected especially in a group which comprised of 48% AA in an underserved area. mAb therapy seems to have a protective effect with significant reduction in the hospitalization and mortality rate among high-risk patients with COVID-19 infection and should be prioritized for administration.

**Disclosures:**

**All Authors**: No reported disclosures

